# Does systemic anti-tumor therapy increase COVID-19 risk in patients with cancer?

**DOI:** 10.1177/10781552211015762

**Published:** 2021-05-07

**Authors:** Murat Ayhan, Şahin Laçin, Deniz T Özyükseler, Heves Sürmeli, Akif Doğan, Merve Turan, Hatice odabas, Nedim Turan, Mahmut Emre Yıldırım

**Affiliations:** 1 Department of Medical Oncology, Kartal Dr. Lütfi Kirdar City Hospital, Health Science University, İstanbul, Turkey; 2 Department of Medical Oncology, Faculty of Medicine, Yeditepe University, İstanbul, Turkey

**Keywords:** COVID-19, cancer, antineoplastic agents, metastatic stage

## Abstract

**Purpose:**

We aimed to determine the COVID-19 infection rate and determine the factors that affect hospitalization and prognosis in patients receiving systemic chemotherapy (CT), immunotherapy (IT) and molecular-targeted therapies at our hospital within three months after the onset of COVID-19 pandemic.

**Materials and methods:**

The patients who received systemic treatment at chemotherapy unit with diagnosis of cancer between 11 March 2020 and 11 June 2020 were included. The clinical and demographic characteristics of patients, the systemic treatments that they received (CT, IT, targeted therapies), and the stage of disease were determined. For the parameters that affect the hospitalization of COVID-19 infected patients were also determined.

**Results:**

Among 1149 patients with cancer, 84 of them were infected with COVID-19, and the median age of infected patients was 61.0 (IQR: 21–84) and 60.7% of them were male. As a subtype of cancers lung cancer was more frequent in the patients who infected with COVID compared with non-infected ones and the difference was statistically significant when the underlying malignities were compared (32.1% vs 19.0%, *p* = 0.031). The hospitalization rate and receiving COVID-19 treatment were more frequent in metastatic patients who were receiving palliative therapy, and the difference was statistically significant (*p* = 0.01, *p* = 0.03). In our study, infection rate was similar among patients treated with CT, IT and CT plus targeted therapy; however, fewer COVID-19 infections were seen at patients who received only targeted therapy.

**Conclusion:**

COVID-19 infection is more frequent in cancer patients and tends to be more severe in metastatic cancer patients receiving anticancer treatment, and the continuation of palliative cancer treatments in these patients may cause increased cancer and infection-related morbidity and mortality.

## Introduction

The coronavirus disease 2019 (COVID-19) announced by World health’s Organization as pandemics has increased the need for intensive care and invasive mechanical ventilator need in cancer patients. It also increased death risk by four to five folds.^1^ Cancer exerts a pressure at the person whom it develops and this condition causes an increase of infection risk in patients with cancer. This risk can increase more with specific oncology therapies (such as chemotherapy (CT), immunotherapy (IT), radiotherapy (RT)). A need for a change in oncology practices has arisen due to these risks. Considering the emergency condition in management of cancer patients, the daily clinical practices have been rearranged.^2,3^ Most of the chemotherapeutic agents prevent blood cell production at bone marrow and can cause anemia, thrombocytopenia and leukopenia/lymphopenia.^4,5^ It was shown that the increased infection risk after chemotherapy depends on this reason.^6,7^ Besides, the gastrointestinal side-effects like nausea, vomiting and poor diet, malnutrition can cause more immunosuppression. As a result, it can bring patients of cancer a sensitive population for the pandemics factor COVID-19 infection. In addition, use of IT in cancer patients during pandemics and discussions on relevant risks are ongoing. In a study, it was determined that intensive care need due to COVID-19 infection in patients with cancer and treated with IT increased, but the mortality did not increase.^8^ Also, some authors mentioned that the T-cell reactivation by IT does not increase COVID-19 infection risk in patients with cancer, it is rather preventive for them.^9^

We aimed to investigate the COVID-19 infection rate and determine hospitalization rate in our patients who were infected after anti-tumor therapy and the factors that affect prognosis in the ones receiving systemic CT, IT and molecular-targeted therapies at our hospital within three months after the onset of COVID-19 pandemic.

## Materials and methods

### Patients

The patients receiving therapy at the chemotherapy unit between 11 March 2020 and 11 June 2020 at Saglık Bilimleri University, Kartal Dr. Lütfi Kırdar City Hospital were determined by screening via hospital information management system. Within this period, 1149 patients with diagnosis of cancer, receiving systemic chemotherapy were detected. The medical oncology clinical follow-up dossiers were reviewed retrospectively and the demographic characteristics, systemic therapies given (such as CT, IT, targeted therapies), the stage of disease were determined. Among patients who receive systemic therapy, the patients who were diagnosed with COVID-19 between 11 March and 11 July 2020 at our hospital were detected via Hospital Information Management System (HIMS). Laboratory findings, treatments and life conditions of these COVID-19 infected patients after follow-ups were determined. The COVID-19 infected patients were separated into two groups as the ones receiving outpatient therapies and inpatients therapies as hospitalized. The monthly number of patients who apply to the medical oncology polyclinic and the rate of chemotherapy given for both 2019 and the period between 11 March and 11 June 2020 were detected. Our study was conducted according to the Principles of Helsinki Declaration and the ethical committee approval was taken from Kartal Dr. Lutfi Kırdar City Hospital Ethical Committee.

### Statistical analysis

SPSS 22.0 (IBM) was used for statistical analysis. The continuous variables were presented as, standard deviation (SD) and the means or quartile range (IQR) and median, appropriately, for descriptive analysis. The numerical variables between two independent conditions were analyzed by Student-t test in case of normal distribution and by Mann-Whitney-u test in case of the opposite. Fisher-exact test was applied to determine the mortality difference between patients with and without cancer. Ki-square test, univariate logistic regression and multivariate logistic regression were used to determine the relationship between clinics plus demographics and mortality. A *p*-value of <0.05 was accepted as statistically significant.

## Results

Eighty-four of 1149 patients with cancer were infected with COVID-19 had a median age of 61.0 (IQR: 21–84) while 1065 patients without COVID-19 infection had a median age of 59.0 (IQR: 22–86); 60.7% of COVID-19 infected patients were male and 39.3% were female; 34.5% of COVID-19 infected patients were still active smokers; but this ratio was 32.7% for patients who were not infected with COVID-19. The most frequent comorbid diseases in patients who were infected with COVID-19 were diabetes mellitus (DM) and hypertension (HT) with 45.7% and 34.3%, respectively. The most frequent comorbidities at non-COVID-19 infected patients were HT and DM with 41.9% and 35.6%, respectively. The rate of lung cancer was 32.1%, breast cancer was 21.4% and the colorectal cancer was the most frequent underlying malignancy for COVID-19 infected patients while the rates were 30.9%, 19.0% and 18.6% for breast, lung and colorectal cancers, respectively, for non-COVID-19 infected patients; 67.9% of COVID-19 infected patients were at metastatic stage while 67.9% were receiving palliative therapy; 77.4% of COVID-19 infected patients received only chemotherapy (CT) as systemic therapy while 15.5% received CT plus molecular targeted therapy. Detailed characteristics of all patients are given at Table 1.

**Table 1.** Patients’ characteristics and their distributions according to COVID negative and positive status.

		COVID (–) 1065 (92.7%)	COVID (+) 84 (7.3%)	
Variable		*n* (%)	*n* (%)	*p*
Age	Median (min–max)	59.0 (22–86)	61.0 (21–84)	
Gender	Female	580 (54.5)	33 (39.3)	**0.007**
	Male	485 (45.5)	51 (60.7)	
Smoking	Current smoker	348 (32.7)	29 (34.5)	0.941
	Non-smoker	445 (41.8)	34 (40.5)	
	Unknown	272 (25.5)	21 (25.0)	
Comorbidity	Hypertension	153 (41.9)	12 (34.3)	
	Diabetes mellitus	130 (35.6)	16 (45.7)	
	Coronary artery disease	47 (12.9)	3 (8.6)	0.504
	COPD	32 (8.8)	3 (8.6)	
	Chronic renal failure	3 (0.8)	1 (2.9)	
Primary malignancy	Breast	329 (30.9)	18 (21.4)	**0.031**
	Lung	202 (19.0)	27 (32.1)	
	Colorectal	198 (18.6)	11 (13.1)	
	Gastric	83 (7.8)	9 (10.7)	
	Ovary	59 (5.5)	1 (1.2)	
	Pancreas	36 (3.4)	4 (4.8)	
	Endometrium	21 (2.0)	3 (3.6)	
	Other	137 (12.9)	11 (13.1)	
Stage	1	14 (1.3)	2 (2.4)	0.381
	2	119 (11.2)	7 (8.3)	
	3	293 (27.5)	18 (21.4)	
	4	639 (60.0)	57 (67.9)	
Patents metastatic disease status during the outbreak	Metastatic	636 (59.7)	57 (67.9)	0.142
	Non-metastatic	429 (40.3)	27 (32.1)	
Treatment Type	Neoadjuvant	102 (9.6)	9 (10.7)	0.222
	Adjuvant	324 (30.4)	18 (21.4)	
	Palliative	639 (60.0)	57 (67.9)	
Treatment line	First-line	411 (30.9)	40 (47.6)	0.414
	Second-line	148 (14.0)	12 (14.3)	
	Third-line	70 (6.6)	5 (6.0)	
Type of anti-cancer therapy	Chemotherapy	728 (68.4)	65 (77.4)	**0.039**
	Targeted-Therapy	176 (16.5)	4 (4.8)	
	CT plus Targeted-Therapy	145 (13.6)	13 (15.5)	
	Immunotherapy	16 (1.5)	2 (2.4)	

CT: chemotherapy; COPD: chronic obstructive pulmonary disease.

The bold written numbers represent statistically significant values.

When patients receiving systemic therapy were compared according to their gender, males were more infected with COVID-19 then females and this difference was statistically significant (60.7% vs 39.3%, respectively; *p* = 0.007). In patients who were both infected with COVID-19 and who did not present the infection, active smoking rates were similar (34.5% vs 32.7%, *p* = 0.941). Lung cancer was more frequent at the patients infected with COVID compared with non-infected ones and this difference was statistically significant when the underlying malignities were compared (32.1% vs 19.0%, *p* = 0.031). The patients who did or did not present the infection were compared by means of their metastatic conditions and no statistical difference was found (67.9% vs 59.7%, *p* = 0.142) (Table 1).

The COVID-19 infected patients were compared according to the underlying malignities and no statistical difference by means of treatment under hospitalization was found (*p* = 0.20). Likewise, receiving COVID-19 under hospitalization did not reveal a significant difference for gender, age and active smoking (*p* = 0.20, *p* = 0.70, *p* = 0.80, respectively). However, metastatic patients and patients receiving palliative therapy among the COVID-19 infected patients were more frequently hospitalized and received COVID-19 treatment, and this difference was statistically significant (*p* = 0.01, *p* = 0.03) (Table 2).

**Table 2.** COVID positive patients’ characteristics and their distributions according to hospitalization or ambulatory status.

Parameter		Hospitalization71 (84.5%)	Ambulatory 13 (15.5%)	*p*-Value
Malignancies	Lung	25 (92.6%)	2 (7.4%)	0.2
	Breast	13 (72.2%)	5 (27.8%)	
	Colorectal	9 (81.8%)	2 (18.2%)	
	Ovarian	1 (100%)	0 (0%)	
	Endometrial	2 (66.7%)	1 (33.3%)	
	Gastric	6 (66.7%)	3 (33.3%)	
	Pancreas	4 (100%)	0 (0%)	
Gender	Female	26 (78.8%)	7 (21.2%)	0.2
	Male	45 (88.2%)	6 (11.8%)	
Age	<60	30 (83.3%)	6 (16.7%)	0.7
	≥60	41 (85.4%)	7 (14.6%)	
Smoking	No	30 (88.2%)	4 (11.8%)	0.1
	Yes	26 (89.7%)	3 (10.3%)	
Stage	Non-metastatic	19 (70.4%)	8 (29.6%)	**0.01**
	Metastatic	52 (91.2%)	5 (8.8%)	
Chemotherapy option	Single agent	27 (87.1%)	4 (12.9%)	0.6
	Combination	44 (83.0%)	9 (17.0%)	
Treatment type	Adjuvant	12 (66.7%)	6 (33.3%)	**0.03**
	Neoadjuvant	7 (77.8%)	2 (22.2%)	
	Palliative	52 (91.2%)	5 (8.8%)	
Radiological involvement	No	1 (100%)	0 (0%)	0.6
	Yes	70 (84.3%)	13 (15.7%)	

**Figure 1.**
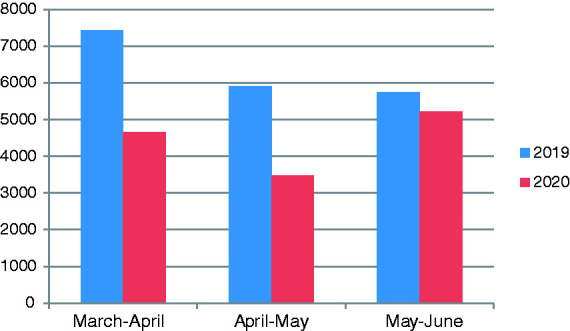
The number of patients who applied to medical oncology polyclinic for 2019 and 2020 (between 11 March and 11 June).

**Figure 2.**
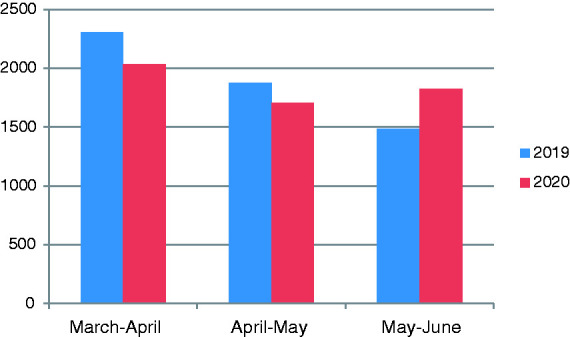
Systemic therapy session given for 2019 and 2020 (between 11 March and 11 June).

The number of patients who applied to medical oncology polyclinic for the period of 11 March–11 June 2020 and the number of patients for the same period of the previous year were compared and analyzed (Table 3). The number of patients who applied to medical oncology polyclinic was compared to the number regarding previous year and a statistical difference was detected after onset of the pandemic, 11 March 2020. It started to increase in June, which is the month that the course of pandemic started to decrease (Figure 1). Very little decrease was found in the number of patients who applied to the chemotherapy unit in order to receive systemic therapy when compared with the number in the previous year (Figure 2).

**Table 3.** Number of patients who applied to the polyclinic between 11 March and 11 June of the years 2019 and 2020 and the session of given systemic therapy.

Number of patients who applied to the polyclinic			Systemic treatment session given	
	2019 (*n*)	2020 (*n*)	2019 (*n*)	2020 (*n*)
March–April	7436	4657	2377	2015
April–May	5909	3481	1965	1819
May–June	5752	5217	1750	2015

## Discussion

Due to the fact that patients with cancer are more exposed to invasive procedures and have to use immunosuppressive agents (like chemotherapy and high-dose steroids) they face more risk of the infection during the COVID-19 pandemic when compared with other people from public. Higher rates of COVID-19 transmission were reported by both patients and healthcare personnel during the pandemic. According to the nationwide statistics made by China Disease Control and Prevention Centre in China, the country where the pandemic was first observed, health institutions reported high risk of COVID-19 transmission.^10^ The high human-to-human transmission rate of COVID-19 has been approved at previous studies on family clusters and travel-related clusters.^11,12^ In Wuhan, 138 patients were evaluated in a retrospective, single-centered study and it was shown that 41% of them had the disease by nosocomial transmission.^13^ At this stage, limiting the hospital control visits of patients, not causing immunosuppression related with the therapy but also not sacrificing from diagnosis, treatment and follow-up become important. Thus, we considered recommendations of ASCO (American Society of Clinical Oncology), ESMO (European Society for Medical Oncology) and Turkish Oncology Society and we continued providing anticancer therapy by preferring the regimens causing less neutropenia risk and administering G-CSF when required in ECOG-PS 0–1 patients with good general conditions. In this study, we investigated COVID-19 rate in patients receiving systemic anti-cancer therapy within the first three months after onset of pandemic. As far as we know, no previous study is present in this topic.

It was shown in the previous studies that the pandemic causes delays in diagnosis and treatment of cancer. In Netherlands, cancer diagnosis decreased by 26% nationwide according to the data obtained from Netherlands Cancer Registry by Din Mohamed et al. between 24 February 2020 and 12 April 2020.^14^ Again, effect of diagnostic delays that will be experienced in four tumor types on cancer survival was guessed in England and authors concluded that an increase is expected in England in the number of preventable deaths due to cancer.^15^ We compared the number of patients who applied to the oncology polyclinic and chemotherapy unit between 11 March and 11 June 2020, with the number of patients of the previous year and the same period; then analyzed the data in order to analyze the effect of pandemic on patients with cancer more comprehensively. Despite the fact that a significant decrease was detected in the number of patients who applied to the medical oncology polyclinic compared with the previous year (Figure 1) a difference was not observed in the number of patients who applied to the chemotherapy unit (Figure 2). It is unclear whether the decrease in applications to the polyclinic during pandemic applied to which patient group and in what ratio; however, considering that no decrease in number of patients who receive chemotherapy was present, we thought that both metastatic and active therapy-receiving patients due to adjuvant purposes continued their therapies despite the pandemics, and also that the metastatic or early-stage patients not receiving active therapy did not visit the hospital.

It was shown that COVID-19 affects men more in the normal population.^16,17^ Also, another study in which 800, COVID-19 infected patients with cancer were examined, 56% of patients were men.^18^ We showed that men experienced more COVID infections after cancer therapy in our study. This difference was statistically significant (*p* = 0.007).

It was shown that patients with lung cancer have more frequent and severe COVID-19 infections due to the decreased lung functions and severe infection in the previous studies.^19–21^ In our study, though patients with breast cancer had more cancer therapy, patients with lung cancer were most frequently infected with COVID-19 (32.1%). This reminds that the cytotoxic agents and the damage in lung can be affecting.

In a multi-centered, observational study performed under the Thoracic Cancers International COVID-19 Collaboration (TERAVOLT), it was reported that 152 of 200 patients with lung cancer (76%) were treated in the hospital and 66 (33%) died in the hospital. Patients who had a surgical operation or chemotherapy were not included in this study. In this study, it was suggested that systemic therapy and immunotherapy did not affect the survival of COVID-19 patients.^22^ Lee et al.^18^ performed a cohort study on 800 cancer patients with COVID-19 and showed that chemotherapy except direct anti-lymphocytic agents and myelosuppressive agents did not increase the susceptibility to infections. In our study, 84 of 1149 cancer patients receiving systemic therapy had COVID-19 infection and 84.5% of them (71) were treated under hospitalization. The net effect of anti-cancer therapies in the first three months at which the pandemic was the most intense on COVID-19 rate is unknown, but the preventive practices applied by hospital managers for cancer patients like social isolation and routine fever measurement prior to therapy, and also the factor that patients and their relatives were aware of the infection could decrease COVID-19 rate among our patients.

Barlesi et al. investigated effects of systemic therapies at cancer patients infected with COVID-19 and observed that chemotherapy received within the last three months caused clinical deterioration mainly on the patients at metastatic stage, while immunotherapy or targeted therapies did not cause such an effect.^23^ In our study, the fact that patients receiving anti-cancer therapy were either metastatic or non-metastatic did not cause a difference in the COVID-19 rate (*p* = 0.142). However, our patients at metastatic stage were hospitalized more due to the infection (*p* = 0.01). This condition can be explained by the fact that metastatic patients can become more fragile with the superseding infection since they were already weak due to bad organ functions and dietary disorders.

Takahari et al.^24^ made recommendations for patients with gastrointestinal cancer during the pandemic according to the severity of pandemic and presence of risks (age > 75, ECOG-PS > 2, comorbidity, etc.). They recommended some changes in the therapy like decreasing intensity of chemotherapy, delaying therapy, decreasing or discontinuing cycles for the places where pandemics transmission rate is high and for the patients under high risk.^24^ Indini et al.^25^ created a risk score to define the cancer patients who are at high risk for COVID-19 infection after a comprehensive literature review. They defined CT, CT + IT, palliative therapy and concurrent steroid therapy as factors that increase the risk by means of COVID-19.^25^ In our study, COVID-19 rate was similar among patients treated with CT, IT and CT plus targeted therapy; however, fewer COVID-19 infections were seen at patients who received only targeted therapy. Similarly, no COVID-19 infection rate difference was observed between the patients receiving either palliative therapy or neoadjuvant or adjuvant therapy.

We count being retrospective, concurrently receiving radiotherapy as the limiting factors in our study. Positive side of this study was evaluated as consisting the patients who received anti-cancer therapy at the first three months which the pandemic was the most intense in our country.

As a result, urgent and population-specific approach is required in the risk of COVID-19 as cancer patients are under more risk and they are more defenseless population compared with the general population. Besides, not delaying cancer therapies of these patients is important and remote evaluation methods and practices like tele-medicine aiming to reduce hospital visits of patients in remission may decrease the infection risk. During the visits, proof of COVID-19 test negativity in both the patient and the person who will accompany especially in cases requiring hospitalization may significantly reduce contamination risk in cancer patients. More long ranging regimens can be recommended instead of weekly regimens in order to decrease the visits of especially patients with metastatic cancer. It is possible to conclude that risks of morbidity and mortality after discontinuation of effective cancer therapies in cancer patients during the pandemic will probably be much more than the risk that will develop due to COVID-19.

## Supplemental Material

sj-pdf-1-opp-10.1177_10781552211015762 - Supplemental material for Does systemic anti-tumor therapy increase COVID-19 risk in patients with cancer? Click here for additional data file. Supplemental material, sj-pdf-1-opp-10.1177_10781552211015762 for Does systemic anti-tumor therapy increase COVID-19 risk in patients with cancer? by Murat Ayhan, Şahin Laçin, Deniz T Özyükseler, Heves Sürmeli, Akif Doğan, Merve Turan, Hatice odabas, Nedim Turan and Mahmut Emre Yıldırım in Journal of Oncology Pharmacy Practice

sj-pdf-2-opp-10.1177_10781552211015762 - Supplemental material for Does systemic anti-tumor therapy increase COVID-19 risk in patients with cancer? Click here for additional data file. Supplemental material, sj-pdf-2-opp-10.1177_10781552211015762 for Does systemic anti-tumor therapy increase COVID-19 risk in patients with cancer? by Murat Ayhan, Şahin Laçin, Deniz T Özyükseler, Heves Sürmeli, Akif Doğan, Merve Turan, Hatice odabas, Nedim Turan and Mahmut Emre Yıldırım in Journal of Oncology Pharmacy Practice

sj-pdf-3-opp-10.1177_10781552211015762 - Supplemental material for Does systemic anti-tumor therapy increase COVID-19 risk in patients with cancer? Click here for additional data file. Supplemental material, sj-pdf-3-opp-10.1177_10781552211015762 for Does systemic anti-tumor therapy increase COVID-19 risk in patients with cancer? by Murat Ayhan, Şahin Laçin, Deniz T Özyükseler, Heves Sürmeli, Akif Doğan, Merve Turan, Hatice odabas, Nedim Turan and Mahmut Emre Yıldırım in Journal of Oncology Pharmacy Practice
