# Supplementary material for: Does systemic anti-tumor therapy increase COVID-19 risk in patients with cancer?
Source: J Oncol Pharm Pract. 2021 May 7;27(6):1461–7. doi: 10.1177/10781552211015762 (PMC8107490; doi:10.1177/10781552211015762)
Supplement: sj-pdf-2-opp-10.1177_10781552211015762 - Supplemental material for Does systemic anti-tumor therapy increase COVID-19 risk in patients with cancer? [file sj-pdf-2-opp-10.1177_10781552211015762.pdf]

Table 1 Patients' characteristics and their distributions according to COVID negative and positive status.				
		COVID (-) 1065 (92.7%)	COVID(+) 84 (7.3%)	P
**Variable**		**n (%)**	**n (%)**	
**Age**	**median (min-max)**	59.0 (22-86)	61.0 (21-84)	
**Gender**	**Female**	580 (54.5)	33 (39.3)	**0.007**
	**Male**	485 (45.5)	51 (60.7)	
**Smoking**	**Current smoker**	348 (32.7)	29 (34.5)	0.941
	**Non-smoker**	445 (41.8)	34 (40.5)	
	**Unknown**	272 (25.5)	21 (25.0)	
**Comorbidity**	**Hypertension**	153 (41.9)	12 (34.3)	
	**Diabetes mellitus**	130 (35.6)	16 (45.7)	
	**Coronary artery disease**	47 (12.9)	3 (8.6)	0.504
	**COPD**	32 (8.8)	3 (8.6)	
	**Chronic renal failure**	3 (0.8)	1 (2.9)	
**Primary malignancy**	**Breast**	329 (30.9)	18 (21.4)	
	**Lung**	202 (19.0)	27 (32.1)	
	**Colorectal**	198 (18.6)	11 (13.1)	
	**Gastric**	83 (7.8)	9 (10.7)	**0.031**
	**Ovary**	59 (5.5)	1 (1.2)	
	**Pancreas**	36 (3.4)	4 (4.8)	
	**Endometrium**	21 (2.0)	3 (3.6)	
	**Other**	137 (12.9)	11 (13.1)	
**Stage**	**1**	14 (1.3)	2 (2.4)	0.381
	**2**	119 (11.2)	7 (8.3)	
	**3**	293 (27.5)	18 (21.4)	
	**4**	639 (60.0)	57 (67.9)	
**Patents metastatic disease status during the outbreak**	**Metastatic**	636 (59.7)	57 (67.9)	0.142
	**Non-metastatic**	429 (40.3)	27 (32.1)	
**Treatment Type**	**Neoadjuvant**	102 (9.6)	9 (10.7)	
	**Adjuvant**	324 (30.4)	18 (21.4)	0.222
	**Palliative**	639 (60.0)	57 (67.9)	
**Treatment line**	**First-line**	411 (30.9)	40 (47.6)	
	**Second-line**	148 (14.0)	12 (14.3)	0.414
	**Third-line**	70 (6.6)	5 (6.0)	
**Type of anti-cancer therapy**	**Chemotherapy**	728 (68.4)	65 (77.4)	
	**Targeted-Therapy**	176 (16.5)	4 (4.8)	
	**CT plus Targeted-Therapy**	145 (13.6)	13 (15.5)	**0.039**
	**Immunotherapy**	16 (1.5)	2 (2.4)	
**CT: Chemotherapy; COPD: Chronic obstructive pulmonary disease**				
