# Supplementary material for: Does systemic anti-tumor therapy increase COVID-19 risk in patients with cancer?
Source: J Oncol Pharm Pract. 2021 May 7;27(6):1461–7. doi: 10.1177/10781552211015762 (PMC8107490; doi:10.1177/10781552211015762)
Supplement: sj-pdf-3-opp-10.1177_10781552211015762 - Supplemental material for Does systemic anti-tumor therapy increase COVID-19 risk in patients with cancer? [file sj-pdf-3-opp-10.1177_10781552211015762.pdf]

**Table 2. COVID positive patients' characteristics and their distributions according to hospitalization or ambulatory status.**				
Parameter		Hospitalization 71 (84.5%)	Ambulatory 13 (15.5%)	P-value
Malignancies	Lung	25 (92.6%)	2 (7.4%)	0.2
	Breast	13 (72.2%)	5 (27.8%)	
	Colorectal	9 (81.8%)	2 (18.2%)	
	Ovarian	1 (100%)	0 (0%)	
	Endometrial	2 (66.7%)	1 (33.3%)	
	Gastric	6 (66.7%)	3 (33.3%)	
	Pancreas	4 (100%)	0 (0%)	
Gender	Female	26 (78.8%)	7 (21.2%)	0.2
	Male	45 (88.2%)	6 (11.8%)	
Age	< 60	30 (83.3%)	6 (16.7%)	0.7
	≥ 60	41 (85.4%)	7 (14.6%)	
Smoking	No	30 (88.2%)	4 (11.8%)	0.1
	Yes	26 (89.7%)	3 (10.3%)	
Stage	Non-metastatic	19 (70.4%)	8 (29.6%)	**0.01**
	Metastatic	52 (91.2%)	5 (8.8%)	
Chemotherapy option	Single agent	27 (87.1%)	4 (12.9%)	0.6
	Combination	44 (83.0%)	9 (17.0%)	
Treatment Type	Adjuvant	12 (66.7%)	6 (33.3%)	**0.03**
	Neoadjuvant	7 (77.8%)	2 (22.2%)	
	Palliative	52 (91.2%)	5 (8.8%)	
Radiological involvement	No	1 (100%)	0 (0%)	0.6
	Yes	70(84.3%)	13 (15.7%)	
